# Perihepatic Adhesions: Another Look

**DOI:** 10.1155/S1064744995000159

**Published:** 1995

**Authors:** Ashwin Chatwani, Nina Mohammed, Soheil Amin-Hanjani, Paul Nyirjesy

**Affiliations:** Department of Obstetrics and Gynecology 7 OPD Temple University Hospital 3401 N. Broad Street Philadelphia PA 19140 USA

## Abstract

*Objective:* The objective of our study was to determine if pelvic inflammatory disease (PID) was the only cause of perihepatic adhesions.

*Methods:* One hundred consecutive patients undergoing elective sterilization by laparoscopy were enrolled in this study. The preoperative workup included a history, physical examination, cervical culture for *Neisseria gonorrhoeae*  and *Chlamydia trachomatis*, leukocyte count, C-reactive protein, and liver-function tests. During the laparoscopic procedure, the pelvis and liver surface were inspected for evidence of any adhesions. If perihepatic adhesions were discovered in a patient without any evidence of prior PID, then cultures from the adhesion, peritoneal fluid, and tubal specimens were obtained for *N. gonorrhoeae, C. trachomatis, Mycoplasma hominis, Ureaplasma urealyticum,* anaerobes, and facultative aerobes. Tubal specimens were also obtained for histologic examination.

*Results:* Of 100 patients, 7 patients had perihepatic adhesions without any laparoscopic evidence of prior PID. The preoperative cultures were negative. Three of these patients had no history of sexually transmitted disease or PID. Their anti-chlamydial antibody titers were also negative. Of the remaining 4 patients with perihepatic adhesions, 2 had a history of gonococcal or chlamydial infection and 2 had histological evidence of chronic salpingitis.

*Conclusions:* The study suggests that PID may not be the only cause of perihepatic adhesions.


Infectious Diseases in Obstetrics and Gynecology 2:263–266 (1995)
© 1995 Wiley-Liss, Inc.

### Perihepatic Adhesions: Another Look

Ashwin Chatwani, Nina Mohammed, Soheil Amin-Hanjani, and Paul Nyirjesy

*Department of Obstetrics and Gynecology, Temple University School of Medicine, Philadelphia, PA*

ABSTRACT

***Objective:*** **The objective of our study was to determine if pelvic inflammatory disease (PID) was the only cause of perihepatic adhesions.**

***Methods:*** **One hundred consecutive patients undergoing elective sterilization by laparoscopy were enrolled in this study. The preoperative workup included a history, physical examination, cervical culture for *Neisseria gonorrhoeae* and *Chlamydia trachomatis*, leukocyte count, C-reactive protein, and liver-function tests. During the laparoscopic procedure, the pelvis and liver surface were inspected for evidence of any adhesions. If perihepatic adhesions were discovered in a patient without any evidence of prior PID, then cultures from the adhesion, peritoneal fluid, and tubal specimens were obtained for *N. gonorrhoeae, C. trachomatis, Mycoplasma hominis, Ureaplasma urealyticum,* anaerobes, and facultative aerobes. Tubal specimens were also obtained for histologic examination.**

***Results:*** **Of 100 patients, 7 patients had perihepatic adhesions without any laparoscopic evidence of prior PID. The preoperative cultures were negative. Three of these patients had no history of sexually transmitted disease or PID. Their anti-chlamydial antibody titers were also negative. Of the remaining 4 patients with perihepatic adhesions, 2 had a history of gonococcal or chlamydial infection and 2 had histological evidence of chronic salpingitis.**

***Conclusions:*** **The study suggests that PID may not be the only cause of perihepatic adhesions.**
© 1995 Wiley-Liss, Inc.

KEY WORDS

Fitz-Hugh-Curtis syndrome, salpingitis, pelvic inflammatory disease

Perihepatic adhesions are generally considered pathognomonic of pelvic inflammatory disease (PID). This extrapelvic manifestation of acute salpingitis is referred to as perihepatitis or The Fitz-Hugh-Curtis syndrome, named after the individuals who first described the syndrome in the United States. In 1919 at a meeting of the Society of Obstetrics and Gynecology in Montevideo, Uruguay, Carlos Stajano[1] presented a case of right-upper-quadrant pain associated with salpingitis. However, it was not until 1930 that Curtis[2] described the chronic aspects of this syndrome in the United States. Four years later, Fitz-Hugh[3] described the acute phase of the syndrome by presenting 3 clinical cases.

The Fitz-Hugh-Curtis syndrome is composed of 2 phases, acute and chronic. The acute phase presents as a sharp, pleuritic-type pain in the right upper quadrant. The pain is usually worsened with coughing, deep inspiration, and movement. An examination of the upper right quadrant either by laparoscopy or laparotomy reveals inflammation of the peritoneum overlying the liver and the anterior abdominal wall. The chronic phase is characterized by typical violin-string adhesions between the anterior abdominal wall and the anterior surface of the liver. The pain associated with perihepatitis usually subsides with medical therapy. Long-term sequelae are rare, but occasionally patients have persistent right-upper-quadrant pain secondary to thick, fi-

Address correspondence/reprint requests to Dr. Ashwin Chatwani, Department of Obstetrics and Gynecology, 7 OPD, Temple University Hospital, 3401 N. Broad Street, Philadelphia, PA 19140.

Received September 26, 1994
Accepted February 20, 1995

Clinical Study brous adhesions. An operative laparoscopy with lysis of adhesions has been described for women with chronic pelvic pain.[4,5] In addition, 2 cases of bowel obstruction in women with perihepatic adhesions have been reported.[6]

A study by Hanjani et al.[7] identified a subgroup of female patients with perihepatic adhesions and no evidence of acute or chronic PID at the time of laparoscopy. This subgroup of patients created the speculation that perihepatic adhesions in women may not solely result from PID. The objective of our study was similar, to determine if PID was the only cause of perihepatic adhesions. In our study, however, more clinical data such as peritoneal-fluid, fallopian-tube, and perihepatic-adhesion cultures were obtained. Additionally, fallopian-tube histology, serum anti-chlamydial antibody titers, erythrocyte sedimentation rate, and C-reactive protein were obtained in an attempt to support in a more rigorous manner the initial conclusion of Hanjani et al.[7]

### SUBJECTS AND METHODS

Prior to initiating the study, we obtained approval from our institutional review board. Enrolled in the study were patients undergoing laparoscopic sterilization. Appropriate consent was obtained from each patient prior to the procedure.

Preoperatively, a complete history was taken to identify previous episodes of sexually transmitted diseases, PID, or endometritis following either vaginal delivery, cesarean delivery, or postabortal dilatation and curettage. The patients were also questioned regarding a history of liver disease and pelvic or abdominal surgery. A complete physical examination was performed to identify significant abdominal or pelvic abnormalities. The preoperative studies included a Papanicolaou smear, cervical cultures for *Chlamydia trachomatis* and *Neisseria gonorrhoeae,* leukocyte count, and serum beta-human chorionic gonadotrophin level.

The laparoscopic tubal ligation was performed by the usual 2-puncture technique using fallope rings. Prior to performance of the tubal ligation, the pelvis was examined for evidence of PID. The laparoscopic criteria considered to be consistent with salpingitis were erythema or swelling of the tubes, pelvic adhesions, evidence of fimbrial occlusion, and purulent discharge. After completion of the tubal ligation, the right upper quadrant was inspected. If perihepatic adhesions were identified in a patient with a normal laparoscopic examination of the pelvis, then additional procedures were performed. Cultures were obtained for *C. trachomatis, Mycoplasma hominis, Ureaplasma urealyticum,* and aerobic/anaerobic organisms from the following sources: peritoneal fluid, biopsy specimens from both fallopian tubes (taken from the knuckle of the tube at the site of fallope-ring placement), and biopsy tissue from perihepatic adhesions. The biopsy specimens from the fallopian tubes, measuring on average 0.5 × 0.5 × 1 cm, were histologically examined for evidence of acute or chronic salpingitis. Serum specimens were sent for erythrocyte sedimentation rate, C-reactive protein, liver transaminases, and anti-chlamydial antibody titers. The most senior author (A.C.) was present as an observer for all cases.

### RESULTS

During a 10-month period, 100 consecutive patients undergoing laparoscopic tubal ligation at Temple University Hospital were enrolled in the study. Seven patients were found to have perihepatic adhesions in the presence of a normal pelvis. Of these 7 patients, 3 had no history of sexually transmitted disease or pelvic infection. Their laboratory studies were all within normal limits (Table 1, patients 1–3).

Of the remaining 4 patients, 2 (Table 1, patients 5 and 7) had evidence of salpingitis by histology. Two patients (Table 1, patients 4 and 5) admitted to having had *N. gonorrhoeae* infection in the past. Two patients (Table 1, patients 4 and 6) had positive serum anti-chlamydial antibody titers. Only 1 patient (Table 1, patient 7) had a positive culture from the fallopian-tube specimens obtained intraoperatively; this organism is rarely isolated from the human genital tract and may represent a contaminant.

### DISCUSSION

Perihepatic adhesions are generally considered pathognomonic of PID. The Fitz-Hugh-Curtis syndrome typically occurs in young, sexually active women. The incidence of this syndrome is variable (4–30%), depending on the criteria used to diagnose perihepatitis and PID.[5,8,9] For many years, *N. gonorrhoeae* was implicated as the organism responsible for this entity. Over the years, other or-

**TABLE 1. Summary of 7 patients with perihepatic adhesions and normal pelvis**

| | Patient No. | | | | | | |
|---|---|---|---|---|---|---|---|
| Patient profile | 1 | 2 | 3 | 4 | 5 | 6 | 7 |
| History of STD[a]/PID | Negative | Negative | Negative | GC[b] | GC | Negative | Negative |
| Preoperative cultures | Negative | Negative | Negative | Negative | Negative | Negative | Negative |
| C-reactive protein/ESR[c] | Normal | Normal | Normal | Normal | Normal | Normal | Normal |
| Liver profile | Normal | Normal | Normal | Normal | Normal | Normal | Normal |
| Histology (fallopian tube): salpingitis | Normal | Normal | Normal | Normal | Mild | Normal | Chronic |
| Chlamydial titer | Negative | Negative | Negative | Positive | Negative | Positive | Negative |
| Peritoneal-fluid culture | Negative | Negative | Negative | Negative | Negative | Negative | Negative |
| Adhesion culture | Negative | Negative | Negative | Negative | Negative | Negative | Negative |
| Fallopian-tube culture | Negative | Negative | Negative | Negative | Negative | Negative | *Pseudomonas providencia* |

[a]STD = sexually transmitted disease.
[b]GC = gonorrhea culture.
[c]ESR = erythrocyte sedimentation rate.

ganisms have been cited in the literature to be associated with PID/perihepatitis. These microorganisms include aerobic/anaerobic organisms, *M. hominis*, *U. urealyticum*, and *C. trachomatis*.

Several routes of dissemination of the microorganisms have been proposed. In the earlier literature, only 1 mode was described: direct extension from the fallopian tubes into the paracolic gutters to the subphrenic space. More recent evidence, such as the finding of perihepatitis in males with current *N. gonorrhoeae* or *C. trachomatis* genital infections, suggests that other routes of dissemination exist. Some proposed mechanisms include transient bacteremia, lymphatic spread, and retroperitoneal spread to the liver surface.[10]

The diagnosis of the Fitz-Hugh-Curtis syndrome depends on a high clinical index of suspicion. In a sexually active patient with symptoms consistent with PID (lower abdominal pain, fever, vaginal discharge, and dyspareunia) and right-upper-abdominal pain, the diagnosis of perihepatitis must be considered. This syndrome is a diagnosis of exclusion; therefore, the more common causes of upper abdominal pain, i.e., hepatitis, cholecystitis, peptic-ulcer disease, and pneumonia, must be ruled out. The onset of right-upper-quadrant pain in patients with the Fitz-Hugh-Curtis syndrome is variable. In a study by Eschenbach[11] in 1984, 60% of the women experienced pain concurrently with their episode of acute salpingitis but 30% developed symptoms 3–10 days after the onset of lower abdominal pain. In the remaining patients, the right-upper-quadrant pain preceded the acute pelvic symptoms by 3–6 days.[11]

Our study has identified a subgroup of patients similar to those described in the original article by Hanjani et al.[7] These patients had perihepatic adhesions without evidence of PID by history, physical examination, laparoscopic examination of the pelvis, cultures, or fallopian-tube histology. The candidates for the study were fertile women undergoing laparoscopic tubal ligation. Of the 100 patients enrolled in the study, 7 patients had perihepatic adhesions without evidence of prior pelvic infection. All of the patients denied previous liver disease, right-upper-quadrant pain, or surgery involving the liver, gallbladder, or biliary tree. Three patients had no clinical or laboratory evidence of PID. Each of the other 4 patients had either a history of sexually transmitted disease, abnormal tubal histology, positive culture data, or serum studies suggesting a previous chlamydial infection. The perihepatic adhesions in all 7 patients seemed similar in terms of quantity and density. Compared with patients whom we have observed with visual evidence of PID on laparoscopy, the adhesions noted in our present study group seemed fewer, finer, and less dense.

In the 3 patients without PID, there could be an alternative explanation for their perihepatic adhesions. Potential causes include subclinical infectious or immunologic processes of the liver that would not have been recognized. As there is no reliable antibody test to detect prior gonococcal infections, they may have had a prior episode of perihepatitis secondary to *N. gonorrhoeae*. However, this last possibility seems less likely, as we feel that, if this were the case, there would be some histological evidence of tubal damage or infection. Another possibility is that the patients had had mild, subclinical non-chlamydial pelvic infections which left them with no histologic or gross evidence of past infection. This last possibility would be, of course, difficult to exclude with the methods of investigation that are presently available. These patients did pass the strict exclusionary criteria which would rule out any obvious cause of right-upper-quadrant pathology including the Fitz-Hugh-Curtis syndrome. The possible limitations of the exclusionary criteria include incomplete or unreliable histories from the patients and the limitations of acquiring or growing culture material.

The absence of apparent PID in the subgroup of 3 patients with perihepatic adhesions is a significant finding in terms of patient evaluation and counseling. For example, infertile patients undergoing diagnostic laparoscopy who are found to have perihepatic adhesions can no longer be assumed to have chronic salpingitis. More work needs to be done to define the cause of perihepatic adhesions in this subgroup of patients.

### REFERENCES

1. Stajano C: La reaction frencia en gynecologica. Sem Med (Buenos Aires) 27:243–247, 1920.
2. Curtis AH: Causes of adhesions in the right upper quadrant. JAMA 94:1221–1223, 1930.
3. Fitz-Hugh T Jr: Acute gonococcal peritonitis of right upper quadrant in women. JAMA 102:2094, 1934.
4. Reichert JA, Valle RF: Fitz-Hugh-Curtis syndrome: A laparoscopic approach. JAMA 236:266–268, 1976.
5. Owens S, Yeko R, Bloy R, Maroulis GB: Laparoscopic treatment of painful perihepatic adhesions in Fitz-Hugh-Curtis syndrome. Obstet Gynecol 78:542–545, 1991.
6. Ross AN, Palanci CK, Jonasson O: Small bowel obstruction as a sequela to Fitz-Hugh-Curtis syndrome. Ill Med J 161:409–413, 1982.
7. Hanjani S, Neely T, Chatwani A: Perihepatic adhesions; not necessarily pathognomonic of pelvic infection. Am J Obstet Gynecol 167:115–117, 1992.
8. Litt IF, Cohen MI: Perihepatitis associated with salpingitis in adolescence. JAMA 240:1253–1255, 1978.
9. Ousrud M: Perihepatitis in pelvic inflammatory disease: Association with intrauterine contraception. Acta Obstet Gynaecol Scand 59:69–73, 1980.
10. Kimiball MW, Knee S: Gonococcal perihepatitis in a male: The Fitz-Hugh-Curtis syndrome. N Engl J Med 282:1082–1083, 1970.
11. Eschenbach DA: Fitz-Hugh-Curtis syndrome. In Holmes KK, Mardh PA, Sparling PF (eds): Sexually Transmitted Diseases. New York; McGraw-Hill International Book Co., pp 633–638, 1984.
